# Phosphorylation of Parkin at Serine65 is essential for activation: elaboration of a Miro1 substrate-based assay of Parkin E3 ligase activity

**DOI:** 10.1098/rsob.130213

**Published:** 2014-03-19

**Authors:** Agne Kazlauskaite, Van Kelly, Clare Johnson, Carla Baillie, C. James Hastie, Mark Peggie, Thomas Macartney, Helen I. Woodroof, Dario R. Alessi, Patrick G. A. Pedrioli, Miratul M. K. Muqit

**Affiliations:** 1 MRC Protein Phosphorylation and Ubiquitylation Unit, University of Dundee, Dundee, UK; 2 Division of Signal Transduction Therapy, University of Dundee, Dundee, UK; 3 College of Medicine, Dentistry and Nursing, University of Dundee, Dundee, UK

**Keywords:** Parkin, PINK1, Miro1, ubiquitin, phosphorylation, Parkinson's disease

## Abstract

Mutations in PINK1 and Parkin are associated with early-onset Parkinson's disease. We recently discovered that PINK1 phosphorylates Parkin at serine65 (Ser^65^) within its Ubl domain, leading to its activation in a substrate-free activity assay. We now demonstrate the critical requirement of Ser^65^ phosphorylation for substrate ubiquitylation through elaboration of a novel *in vitro* E3 ligase activity assay using full-length untagged Parkin and its putative substrate, the mitochondrial GTPase Miro1. We observe that Parkin efficiently ubiquitylates Miro1 at highly conserved lysine residues, 153, 230, 235, 330 and 572, upon phosphorylation by PINK1. We have further established an E2-ubiquitin discharge assay to assess Parkin activity and observe robust discharge of ubiquitin-loaded UbcH7 E2 ligase upon phosphorylation of Parkin at Ser^65^ by wild-type, but not kinase-inactive PINK1 or a Parkin Ser65Ala mutant, suggesting a possible mechanism of how Ser^65^ phosphorylation may activate Parkin E3 ligase activity. For the first time, to the best of our knowledge, we report the effect of Parkin disease-associated mutations in substrate-based assays using full-length untagged recombinant Parkin. Our mutation analysis indicates an essential role for the catalytic cysteine Cys431 and reveals fundamental new knowledge on how mutations may confer pathogenicity via disruption of Miro1 ubiquitylation, free ubiquitin chain formation or by impacting Parkin's ability to discharge ubiquitin from a loaded E2. This study provides further evidence that phosphorylation of Parkin at Ser^65^ is critical for its activation. It also provides evidence that Miro1 is a direct Parkin substrate. The assays and reagents developed in this study will be important to uncover new insights into Parkin biology as well as aid in the development of screens to identify small molecule Parkin activators for the treatment of Parkinson's disease.

## 2. Introduction

Parkinson's disease is an incurable neurodegenerative disorder whose incidence is set to rise in the forthcoming decades [1]. Over the past 16 years, spectacular genetic breakthroughs have uncovered nearly 20 genes or loci associated with familial Parkinson's disease (PD) that provide a solid biochemical platform to uncover the molecular origins and mechanisms underlying this devastating disorder [2].

Mutations in the RING-IBR-RING (RBR) ubiquitin E3 ligase Parkin were first identified in 1998 in families with early-onset autosomal-recessive PD [3]. Parkin is a 465 amino acid enzyme comprising: a regulatory Ubl domain (residues 1–76); a RING0 domain (residues 145–215); a RING1 domain (residues 237–292) that binds to an E2; an IBR domain (residues 327–378); and a RING2 domain that mediates the enzyme's catalytic activity (415–465) [4]. Recent groundbreaking insights have revealed that Parkin and other members of the RBR family of E3 ligases exhibit HECT-like properties [5,6]. Specifically, Parkin contains a highly conserved catalytic cysteine (Cys^431^) within its RING2 domain, which acts as a ubiquitin acceptor that forms an intermediate thioester bond prior to ubiquitylation of its substrate [5]. The physiological relevance of this catalytic cysteine is underscored by the presence of a human disease-causing mutation at this residue (Cys431Phe), which has been shown to abolish Parkin catalytic activity at least in auto-ubiquitylation assays [7–11].

Historically, Parkin was thought to be constitutively active, but in 2011 it was demonstrated that Parkin's E3 ligase activity is regulated by an interaction between the N-terminal Ubl domain and the C-terminus of the protein, which maintains the enzyme in an autoinhibited closed conformation [12]. The N-terminal Ubl domain plays a critical role in mediating this autoinhibition, because removal of the Ubl domain led to constitutive activation of Parkin [12,13]. Furthermore, expression of Parkin with epitope tags fused to the N-terminus leads to disruption of the Ubl-mediated autoinhibition and activation of E3 ligase activity [12,13]. It is therefore critical to study the properties of recombinant Parkin using full-length protein that is devoid of epitope tags. The physiological relevance of Ubl-mediated autoinhibition is also emphasized by the discovery that PTEN-induced kinase 1 (PINK1), mutations of which also lead to familial PD [14], phosphorylates Parkin at a highly conserved residue Serine65 (Ser^65^) that lies within the Ubl domain; and that phosphorylation leads to activation of Parkin E3 ligase activity as judged by the formation of free ubiquitin chains in a substrate-free ubiquitylation assay [15]. In agreement with our initial findings, several laboratories have reproduced PINK1-dependent phosphorylation of Parkin at Ser^65^ [7,16,17].

The direct regulation of Parkin by PINK1 is consistent with previous clinical and genetic studies that have suggested that both these enzymes function in a common pathway. PINK1 and Parkin patients share a similar phenotype comprising early age at onset, slow progression, dystonia and early development of l-DOPA-induced dyskinesias [18,19]. In addition, studies in *Drosophila melanogaster* provided genetic evidence that PINK1 and Parkin are linked, because PINK1 and Parkin null flies exhibit near identical phenotypes, including mitochondrial deficits, flight muscle degeneration and motor deficits [20–22]. Moreover, overexpression of Parkin can rescue PINK1 null flies, but the opposite is not the case, providing genetic evidence that PINK1 acts upstream of Parkin [20–22]. An upstream role for mammalian PINK1 had also been suggested by cellular studies reporting that PINK1 was required for Parkin recruitment to mitochondria following depolarization of the mitochondrial membrane potential [23–26].

Recently, a low-resolution X-ray crystal structure of full-length rat Parkin and high-resolution rat and human structures missing the Ubl domain have been solved which confirm that Parkin exists in an autoinhibited conformation [27–29]. The full-length structure reveals that autoinhibition of Parkin is mediated by an interaction between the Ubl domain, regulatory element of Parkin (REP helix) and the RING1 domain, obscuring the potential E2 binding site. Direct interaction of the REP helix, that lies between the IBR and RING2 domain, with RING1 domain was also confirmed in human Parkin structures lacking the N-terminal Ubl domain [27,28]. In addition, a further autoinhibitory interaction between the RING0 and RING2 domains which occludes the catalytic Cys^431^ was observed [4,27–29]. However, the structures do not provide any mechanistic insights into how phosphorylation at Ser^65^ mediates transition from an inactive to an active conformation.

An outstanding question in the field is whether Ser^65^ phosphorylation of Parkin is critical for its ability to ubiquitylate substrates. The list of reported potential Parkin substrates is considerable and continues to grow with over 100 suggested [30–35]. However, in the majority of the previous work, experiments have been undertaken using overexpression approaches using Parkin with activating N-terminal tags or Parkin lacking its autoinhibitory Ubl domain containing the PINK1 phosphorylation motif. Much more work is therefore needed to establish whether ubiquitylation of all of the proposed substrates at the level of the endogenous protein is indeed mediated by Parkin. Such validation is important as it will enable identification of the crucial Parkin substrates that determine survival of dopaminergic neurons in Parkinson's disease [33,36–38].

Several lines of evidence indicate that physiological substrates of Parkin reside in the mitochondria, including the observation of mitochondrial deficits in Parkin knockout (KO) mice [39,40] and *Drosophila* models [20–22]; and cellular studies linking Parkin to the regulation of mitochondrial dynamics, turnover and transport [36,37,41,42]. Recently, Miro1, an atypical mitochondrial GTPase, has emerged as a candidate Parkin substrate based on genetic interaction data in *Drosophila* models of Parkin [43] and overexpression studies of N-terminal-tagged mammalian Parkin [32,43,44].

In this paper, we investigate whether Parkin phosphorylation at Ser^65^ is required for its catalytic activation and ubiquitylation of substrates. We demonstrate that Parkin, upon phosphorylation at Ser^65^, can ubiquitylate Miro1 in addition to catalysing the formation of free ubiquitin chains and this is abolished by deletion of the Ubl domain. We have mapped the major sites of Miro1 ubiquitylation to highly conserved Lysine153 (Lys^153^), Lysine230 (Lys^230^), Lysine235 (Lys^235^), Lysine330 (Lys^330^) and Lysine572 (Lys^572^) residues. Using this novel assay, we have undertaken an E2 scan and observed 23/25 E2 ligases that enable Parkin phosphorylated at Ser^65^ to ubiquitylate Miro1. Furthermore, we have deployed our assay to investigate the effect of disease-associated point mutations of Parkin and discovered diverse effects of mutations on Parkin E3 ligase activity, including the identification of several mutants that disrupt the formation of free ubiquitin chains without any significant impact on Miro1 substrate ubiquitylation.

To gain further mechanistic insights into the effect of PINK1 phosphorylation at Ser^65^ on Parkin E3 ligase activity, we have developed a ubiquitin discharge assay, which measures the ability of Parkin to stimulate the discharge of ubiquitin from the E2 ligase UbcH7. We observe that only upon phosphorylation of Parkin at Ser^65^ can it lead to efficient discharge of UbcH7 loaded with ubiquitin. We have used this assay to study the effect of Parkin disease mutations and uncover several mutants that disrupt Ser^65^-phosphorylated Parkin-mediated E2 discharge, shedding light on how these mutations may lead to reduced Parkin-E3-mediated ubiquitylation.

This study validates the critical role of Ser^65^ phosphorylation in enabling Parkin activation of its E3 ligase activity and reveals new mechanistic insights into how disease-associated mutations of Parkin may impact on E3 ligase activity. The assays and technologies described in this study have enabled a more accurate assessment of Parkin E3 ligase activity and could also be deployed in future chemical screening programmes to develop small molecule activators of Parkin for the treatment of Parkinson's disease.

## 3. Material and methods

### 3.1. Materials

[γ-^32^P] ATP was from Perkin-Elmer. All mutagenesis was carried out using the QuikChange site-directed mutagenesis method (Stratagene) with KOD polymerase (Novagen). All DNA constructs were verified by DNA sequencing, which was performed by The Sequencing Service, School of Life Sciences, University of Dundee, using DYEnamic ET terminator chemistry (Amersham Biosciences) on automated DNA sequencers (Applied Biosystems). DNA for bacterial protein expression was transformed into *Escherichia coli* BL21 DE3 RIL (codon plus) cells (Stratagene). All cDNA plasmids, antibodies and recombinant proteins generated for this study are available on request through our reagents website (http://mrcppureagents.dundee.ac.uk/).

### 3.2. Antibodies

Antigen affinity-purified sheep anti-SUMO-1 antibody was a kind gift from Professor Ron Hay (Dundee). Anti-Parkin mouse monoclonal was obtained from Santa Cruz Biotechnology; anti-FLAG HRP-conjugated antibody was obtained from Sigma; anti-maltose binding protein (MBP) HRP-conjugated antibody was obtained from New England Biolabs.

### 3.3. Immunoblotting

Samples were subjected to SDS/PAGE (8–14%) and transferred onto nitrocellulose membranes. Membranes were blocked for 1 h in Tris-buffered saline with 0.1% Tween (TBST) containing 5% (w/v) non-fat dried skimmed milk powder. Membranes were probed with the indicated antibodies in TBST containing 5% (w/v) non-fat dried skimmed milk powder for 1 h at room temperature. Detection was performed using HRP-conjugated antibodies and enhanced chemiluminescence reagent.

### 3.4. *In vitro* ubiquitylation assays

Wild-type or indicated mutant Parkin (2 μg) was initially incubated with 1 μg (or indicated amounts) of *E. coli*-expressed wild-type or kinase-inactive (D359A) MBP-TcPINK1 in a reaction volume of 25 μl (50 mM Tris–HCl (pH 7.5), 0.1 mM EGTA, 10 mM magnesium acetate, 1% 2-mercaptoethanol and 0.1 mM ATP. Kinase assays were incubated at 30°C for 60 min followed by addition of ubiquitylation assay components and Mastermix to a final volume of 50 μl (50 mM Tris–HCl (pH 7.5), 0.05 mM EGTA, 10 mM MgCl_2_, 0.5% 2-mercaptoethanol, 0.12 μM human recombinant E1 purified from Sf21 insect cell line, 1 μM human recombinant UbcH7 and 2 μg 6xHis-Sumo-Miro1 (wild-type or point mutants) both purified from *E. coli*, 0.05 mM Flag-ubiquitin (Boston Biochem) and 2 mM ATP). Ubiquitylation reactions were incubated at 30°C for 60 min and terminated by addition of SDS sample buffer. For all assays, reaction mixtures were resolved by SDS–PAGE. Ubiquitylation reactions were subjected to immunoblotting with anti-FLAG antibody (Sigma, 1 : 7500), anti-Parkin, anti-SUMO1 or anti-MBP antibodies. For the E2 scan, a version of the E2^scan^ kit was obtained from Ubiquigent, and 1 μg of each E2 enzyme was used per reaction.

### 3.5. *In vitro* E2 discharge assays

Wild-type or indicated mutant Parkin (2 μg) was incubated with 1 μg of *E. coli*-expressed wild-type or kinase-inactive (D359A) MBP-TcPINK1 in a reaction volume of 15 μl (50 mM HEPES (pH 7.5), 0.1 mM EGTA, 10 mM magnesium acetate and 0.1 mM ATP). Kinase assays were incubated at 30°C for 60 min. E2-charging reaction was assembled in parallel in 5 μl containing Ube1 (0.5 μg), an E2 (2 μg), 50 mM HEPES pH 7.5 and 10 μM ubiquitin in the presence of 2 mM magnesium acetate and 0.2 mM ATP. After initial incubation of 60 min at 30°C, the reactions were combined and allowed to continue for a further 15 min or indicated times at 30°C. Reactions were terminated by the addition of 5 μl of LDS loading buffer and subjected to SDS–PAGE analysis in the absence of any reducing agent. Gels were stained using InstantBlue.

### 3.6. In-solution protein digestion

*In vitro* ubiquitylation assays were terminated with 1% Rapigest and reduced in 5 mM Tris-(2-carboxyethyl)phosphine (TCEP) at 50°C for 30 min. Additional Tris–HCl was added to 100 mM to ensure buffering at pH 7.5 followed by cysteine alkylation in 10 mM chloroacetamide at 20°C in the dark for 30 min. Samples were diluted to 0.1% Rapigest and digested with 1 : 50 w/w trypsin overnight at 37°C. Peptides were acidified with 1% trifluoroacetic acid and incubated at 37°C for 1 h before precipitating acid-cleaved Rapigest by centrifugation at 17 000*g* for 10 min. Peptides were purified on C18 MicroSpin columns (The Nest Group) before MS analysis. Approximately 30 ng of peptide was analysed by C18 LC–MS/MS over a 60 min gradient from 1% to 37% acetonitrile/0.1% formic acid. Mass spectrometric analysis was conducted by data-dependent acquisition with spectra acquired by collision-induced dissociation on an LTQ-Orbitrap Velos (Thermo Fisher Scientific). Data were analysed using Mascot (www.matrixscience.com), and ion signals were extracted using Skyline [45].

### 3.7. In-gel protein digestion

Protein bands were excised from the gel and washed sequentially with 0.5 ml of water, 50% acetonitrile, 0.1 M NH_4_HCO_3_ and 50% acetonitrile/50 mM NH_4_HCO_3_. All washes were performed for 10 min on a Vibrax shaking platform. Proteins were then reduced with 10 mM DTT/0.1 M NH_4_HCO_3_ at 65°C for 45 min and alkylated with 50 mM chloroacetamide/0.1 M NH_4_HCO_3_ for 20 min at room temperature. They were then washed with 0.5 ml 50 mM NH_4_HCO_3_ and 50 mM NH_4_HCO_3_/50% acetonitrile (as before). Gel pieces were shrunk with 0.3 ml acetonitrile for 15 min. Acetonitrile was aspirated, and trace amounts were removed by drying sample in a Speed-Vac. Gel pieces were then incubated for 16 h with 5 μg ml^−1^ trypsin in 25 mM triethylammonium bicarbonate at 30°C on a shaker. An equal volume of acetonitrile (same as trypsin) was added to each sample and further incubated on a shaking platform for 15 min. The supernatants were dried by Speed-Vac. Another extraction was performed by adding 100 μl 50% acetonitrile/2.5% formic acid for 15 min. This supernatant was combined with the first extract and dried by Speed-Vac. Peptides were purified on C18 MicroSpin columns (The Nest Group) before MS analysis as described for in-solution protein digestions.

### 3.8. Kinase assays

Reactions were set up in a volume of 25 μl, using 2 μg of wild-type or indicated mutants of Parkin and 1 μg of *E. coli*-expressed wild-type or kinase-inactive (D359A) MBP-TcPINK1, in 50 mM Tris–HCl (pH 7.5), 0.1 mM EGTA, 10 mM MgCl_2_, 2 mM DTT and 0.1 mM [γ-^32^P] ATP (approx. 500 cpm pmol^−1^). Assays were incubated at 30°C with shaking at 1050 r.p.m. and terminated after 60 min by addition of SDS sample loading buffer. The reaction mixtures were then resolved by SDS–PAGE. Proteins were detected by Coomassie staining, and gels were imaged using an Epson scanner and dried completely using a gel dryer (Bio-Rad). Incorporation of [γ-^32^P] ATP into substrates was analysed by autoradiography using Amersham hyperfilm.

### 3.9. Buffers for *Escherichia coli* protein purification

For Parkin purification: lysis buffer contained 50 mM Tris–HCl (pH 7.5), 150 mM NaCl, 1 mM EDTA, 1 mM EGTA, 5% (v/v) glycerol, 1% (v/v) Triton X-100, 0.1% (v/v) 2-mercaptoethanol, 1 mM benzamidine and 0.1 mM PMSF. Wash buffer contained 50 mM Tris–HCl (pH 7.5), 500 mM NaCl, 0.1 mM EGTA, 5% (v/v) glycerol, 0.03% (v/v) Brij-35, 0.1% (v/v) 2-mercaptoethanol, 1 mM benzamidine and 0.1 mM PMSF. Equilibration buffer contained 50 mM Tris–HCl (pH 7.5), 150 mM NaCl, 0.1 mM EGTA, 5% (v/v) glycerol, 0.03% (v/v) Brij-35, 0.1% (v/v) 2-mercaptoethanol, 1 mM benzamidine and 0.1 mM PMSF. Elution buffer was equilibration buffer with the addition of 12 mM maltose. Storage buffer was equilibration buffer with the addition of 0.27 M sucrose, and glycerol–PMSF and benzamidine were omitted.

### 3.10. Protein purification from *Escherichia coli*

Full-length wild-type and kinase-inactive TcPINK1 was expressed in *E. coli* as MBP fusion protein and purified as described previously [15]. Briefly, BL21 codon + transformed cells were grown at 37°C to an OD_600_ of 0.3, then shifted to 16°C and induced with 250 μM isopropyl β-d-thiogalactoside (IPTG) at OD_600_ of 0.5. Cells were induced with 250 μM IPTG at OD 0.6 and were further grown at 16°C for 16 h. Cells were pelleted at 4000 r.p.m., and then lysed by sonication in lysis buffer. Lysates were clarified by centrifugation at 30 000*g* for 30 min at 4°C followed by incubation with 1 ml per litre of culture of amylose resin for 1.5 h at 4°C. The resin was washed thoroughly in wash buffer, then equilibration buffer, and proteins were then eluted. Proteins were dialysed overnight at 4°C into storage buffer, snap-frozen and stored at −80°C until use.

Wild-type and indicated mutant untagged Parkin (His-SUMO cleaved) was expressed and purified using a modified protocol [12]. We did not observe any significant difference in solubility or expression between the mutants and wild-type Parkin protein. BL21 cells were transformed with His-SUMO-tagged Parkin constructs, overnight cultures were prepared and used to inoculate 12 × 1l LB medium, 50 µg ml^−1^ carbenicillin, 0.25 mM ZnCl_2_. The cells were grown at 37°C until the OD_600_ was 0.4, and the temperature was dropped to 16°C. At OD_600_ = 0.8, expression was induced with 10 µM IPTG. After overnight incubation, the cells were collected and lysed in 75 mM Tris pH 7.5, 500 mM NaCl, 0.2% Triton X-100, 25 mM imidazole, 0.5 mM TCEP, 1 mM pefablok, 10 µg ml^−1^ leupeptin. After sonication and removal of insoluble material, His-SUMO-Parkin was purified via Ni^2+^–NTA–sepharose chromatography. The protein was collected by elution with 400 mM imidazole in 50 mM Tris, pH 8.2, 200 mM NaCl, 10% glycerol, 0.03% Brij-35, 0.5 mM TCEP. This was dialysed twice against 50 mM Tris pH 8.2, 200 mM NaCl, 10% glycerol, 0.5 mM TCEP in the presence of His-SENP1 415–643 at a ratio of 1 mg His-SENP1 per 5 mg His-SUMO-Parkin. The protease, the His-SUMO tag and any uncleaved protein were removed by two subsequent incubations with Ni^2+^–NTA–sepharose. The cleaved Parkin was further purified in 50 mM Tris, pH 8.2, 200 mM NaCl, 20% glycerol, 0.03% (v/v) Brij-35, 0.5 mM TCEP over a Superdex 200 column.

Wild-type 6xHis-Sumo-Miro1 (1–592), K572R and K567R mutants were expressed in *E. coli*. Briefly, BL21 CodonPlus (DES)-RIL-transformed cells were grown at 37°C to an OD_600_ of 0.4, then reduced to 15°C and induced with 10 μM IPTG at an OD_600_ of 0.6. Cells were then grown at 15°C for a further 20 h. Cells were pelleted at 4200*g* and then lysed by sonication in lysis buffer. Lysates were clarified by centrifugation at 30 000*g* for 30 min at 4°C followed by incubation with Cobalt resin at 4°C for 45 min. The resin was washed thoroughly in high salt buffer, then equilibrated in low salt buffer, and the proteins were then eluted. The eluted Miro1 proteins were further purified by anion exchange chromatography. Proteins were applied to a Mono-Q HR 5/5 column and chromatographed with a linear gradient of NaCl from 0 to 0.5 M. Fractions containing the purified Miro1 protein were then dialysed, snap-frozen in liquid nitrogen and stored at −70°C.

## 4. Results

### 4.1. Ubiquitylation of Miro1 by Parkin is dependent on phosphorylation at Ser^65^

We previously reported the striking observation that untagged full-length recombinant Parkin expressed in *E. coli* was able to induce formation of low-molecular-weight free ubiquitin chains in a substrate-free ubiquitylation assay following phosphorylation of Parkin at Ser^65^ by the active insect orthologue of PINK1, *Tribolium castaneum* (TcPINK1) [15]. To obtain further evidence that PINK1 phosphorylation activates Parkin and to develop a more robust *in vitro* Parkin assay, we tested whether ubiquitylation of the proposed direct substrate Miro1 [32,43,44] could be deployed to assess Parkin activity. We were unable to express full-length recombinant Miro1 (residues 1–618) in *E. coli*, but a fragment of Miro1 (residues 1–592) lacking the C-terminal transmembrane domain expressed well and was used in subsequent assays.

**Figure 1.**
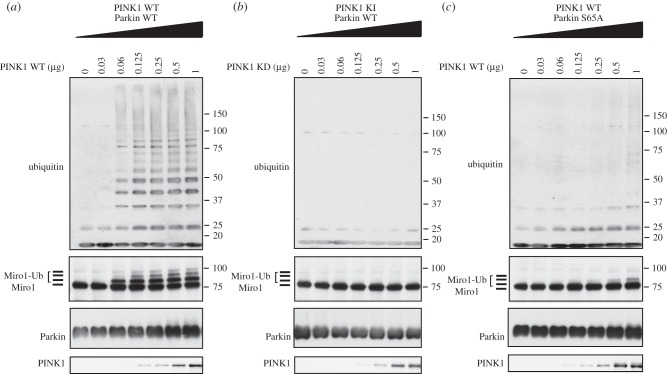
PINK1-dependent phosphorylation of Parkin Ser^65^ leads to activation of Parkin E3 ligase activity and multi-monoubiquitylation of Miro1. Wild-type (WT) (*a*) but not kinase-inactive (KI) (*b*) PINK1 activates wild-type Parkin E3 ligase activity leading to Miro1 multi-monoubiquitylation, an effect that is blocked by mutant Parkin Ser65Ala (S65A) (*c*). Two micrograms of wild-type or S65A Parkin were incubated with indicated amounts of wild-type or kinase-inactive (D359A) MBP-TcPINK in a kinase reaction (50 mM Tris–HCl (pH 7.5), 0.1 mM ethylene glycol tetra-acetic acid (EGTA), 10 mM MgCl_2_, 0.1% 2-mercaptoethanol and 0.1 mM ATP) for 60 min. The ubiquitylation reaction was then initiated by addition of ubiquitylation assay components (50 mM Tris–HCl (pH 7.5), 0.05 mM EGTA, 10 mM MgCl_2_, 0.5% 2-mercaptoethanol, 0.12 μM human recombinant E1 purified from Sf21 insect cell line, 1 μM human recombinant UbcH7 purified from *E. coli*, 0.05 mM Flag-ubiquitin (Boston Biochem) and 2 mM ATP) and 2 μg of His-Sumo-Miro1. Reactions were terminated after 60 min by addition of SDS–PAGE loading buffer and resolved by SDS–PAGE. Miro1, ubiquitin, Parkin and PINK1 were detected using anti-SUMO, anti-FLAG, anti-Parkin and anti-MBP antibodies, respectively. Representative of three independent experiments.

The maximal stoichiometry of Parkin phosphorylation by PINK1 under our assay conditions is 0.08 moles phosphate per mole of protein (the electronic supplementary material, figure S1). To assess whether phosphorylation of Parkin by PINK1 influenced its ability to ubiquitylate Miro1, we phosphorylated untagged full-length Parkin with increasing levels of TcPINK1 in the presence of ATP and then added a reaction mix containing E1 ubiquitin-activating ligase, UbcH7 conjugating E2 ligase, ubiquitin, Mg-ATP and Miro1(1–592). After 60 min, reactions were terminated with SDS sample buffer in the presence of 2-mercaptoethanol and heated at 100°C, and substrate ubiquitylation was assessed by immunoblot analysis with antibodies that detect ubiquitin, Parkin, Miro1 and TcPINK1. Consistent with previous findings in the absence of PINK1 phosphorylation, Parkin was inactive as no evidence of free ubiquitin chain formation or Miro1 ubiquitylation was observed (figure 1*a*, lane 1); with the addition of wild-type TcPINK1, Miro1 multi-monoubiquitylation (a major mono- and minor multi-ubiquitylated species) in addition to free polyubiquitin chain formation was observed (figure 1*a*, lane 3–7). No significant Miro1 ubiquitylation or polyubiquitin chain formation was observed in the presence of the kinase-inactive TcPINK1 (figure 1*b*) or using the Ser65Ala (S65A) Parkin point mutant (figure 1*c*), indicating that Miro1 ubiquitylation is dependent on Parkin Ser^65^ phosphorylation. Using mass spectrometry, we detected the formation of diverse ubiquitin–ubiquitin linkages, including K6, K11, K33, K48 and K63 in reactions containing activated Parkin (the electronic supplementary material, figure S2). We also detected K27 chains, but these were generated in a Parkin-independent manner (the electronic supplementary material, figure S2).

### 4.2. The Ubl domain of Parkin is required for Miro1 substrate ubiquitylation

**Figure 2.**
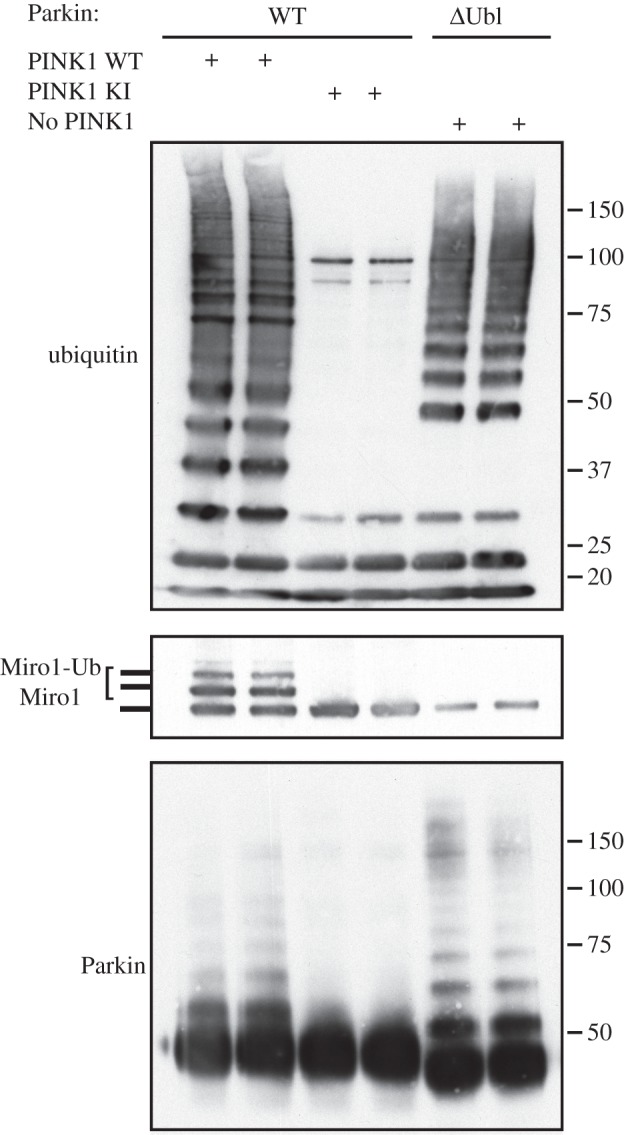
Ubl domain of Parkin is necessary for substrate ubiquitylation. Full-length (lanes 1,2), but not ΔUbl-Parkin (lanes 5,6) ubiquitylates Miro1. Full-length (WT) Parkin was incubated in presence of wild-type (WT) or kinase-inactive (KI) PINK1 as described previously alongside ΔUbl-Parkin in the absence of PINK1. Reactions were analysed by SDS–PAGE; Miro1, ubiquitin and Parkin were detected using anti-SUMO, anti-FLAG and anti-Parkin antibodies, respectively.

To further investigate the role of the Ubl domain in Parkin-mediated Miro1 ubiquitylation, we expressed a fragment lacking the Ubl domain (residues 80–465, ΔUbl-Parkin) and assayed it in parallel with full-length Parkin pre-incubated with either wild-type or kinase-inactive TcPINK1. While ΔUbl-Parkin exhibited significant auto-ubiquitylation activity similar to activated full-length Parkin, it could not catalyse Miro1 ubiquitylation or the formation of low molecular weight polyubiquitin chains (figure 2).

### 4.3. Identification of Parkin-mediated Miro1 ubiquitylation sites

**Figure 3.**
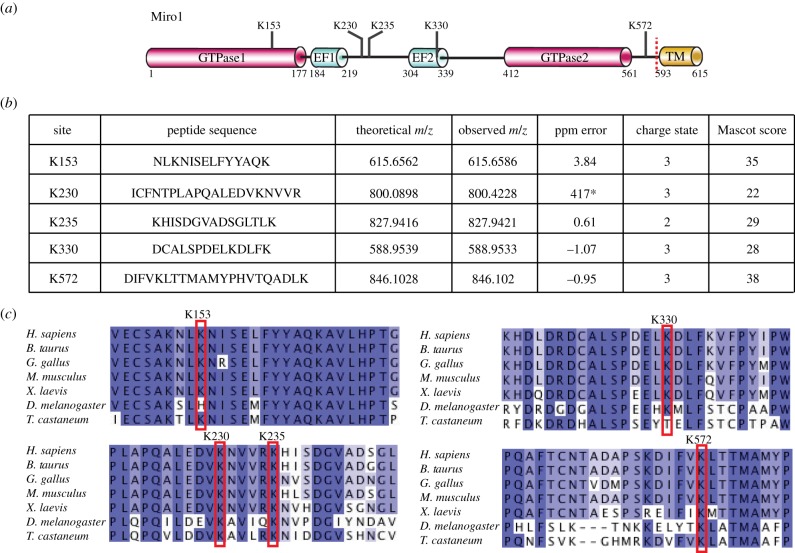
Parkin ubiquitylates Miro1 at multiple sites in a PINK1-dependent manner. (*a*) A schematic of Miro1 domain architecture showing the identified ubiquitylation sites and truncation site (red dotted line) used in this paper. (*b*) Identification of Lys^153^, Lys^230^, Lys^235^, Lys^330^ and Lys^572^ ubiquitylation sites on Miro1. Ubiquitylation assays using wild-type (WT) and kinase-inactive (KI) PINK1 (D359A) in combination with WT Parkin and the substrate Miro1 were undertaken as described in §3. The SDS–PAGE bands were subjected to in-gel tryptic digestion and analysis by LTQ-Orbitrap mass spectrometry. Ubiquitin isopeptides were identified by Mascot (www.matrixscience.com), and spectra were manually validated to ensure peptide fragmentation gave good sequence coverage (*417 ppm error equates to a −1.81 ppm error around the C13 isotope). (*c*) Sequence alignment of residues around Lys^153^, Lys^230^ and Lys^235^ (left), Lys^330^ and Lys^572^ (right), respectively, in human Miro1 and a variety of lower organisms, showing high degree of conservation.

We next sought to determine the major site(s) of Miro1 ubiquitylation by Parkin that had been activated by PINK1 phosphorylation. *In vitro* ubiquitylation of Miro1 by untagged Parkin pre-incubated with wild-type or kinase-inactive TcPINK1 was conducted as described above, followed by in-gel tryptic digestion. Mass spectrometric analysis was conducted as described in §3. This analysis resulted in the identification of five peptides carrying a Gly–Gly ubiquitin tryptic remnant in Parkin activated by wild-type TcPINK1, but which were not seen in corresponding samples of Parkin in the presence of kinase-inactive TcPINK1 (figure 3*a–c*). Lys^153^ was found in a tryptic peptide, located within the first GTPase domain; Lys^230^ and Lys^235^ were found carrying di-Gly remnants in two independent peptides found within the central linker region; and Lys^330^ was identified in the fourth peptide located within the second EF-hand domain of Miro1 (figure 3*a–c*). A fifth peptide containing a di-Gly remnant at Lys^572^ was located within the C-terminal non-catalytic region of Miro1 between the second GTPase domain and the transmembrane domain (figure 3*a–c*). Parallel in-solution tryptic digestion and analysis also identified a miscleaved peptide,MPPPQAFTCNTADAPSKDIFVK(GG)LTTMAMYPHVTQAD LK, spanning Lys^572^ and another highly conserved lysine residue, Lys^567^ (data not shown). Peptide fragmentation pattern analysis supported the modification occurring at Lys^572^. To confirm this we undertook mutagenesis analysis, which revealed that a single Lys572Arg point mutant of Miro1(1–592) significantly reduced the major band of monoubiquitylation, and reduced the minor bands of multi-monoubiquitylation by Parkin phosphorylated by PINK1 (the electronic supplementary material, figure S3). In contrast, mutation of the Lys^567^ residue had no effect on ubiquitylation of Miro1 (the electronic supplementary material, figure S3). All sites identified are highly conserved (figure 3*c*), and these analyses indicate that Miro1 undergoes multi-monoubiquitylation and that Lys^153^, Lys^230^, Lys^235^, Lys^330^ and Lys^572^ are the major sites of Miro1 ubiquitylation targeted by activated Parkin. Several of these residues in Miro1 (Lys^153^, Lys^235^ and Lys^572^) were also recently reported to be ubiquitylated *in vivo* in cells overexpressing tagged Parkin [32].

### 4.4. E2s exhibit differential effects on Parkin-mediated ubiquitylation

**Figure 4.**
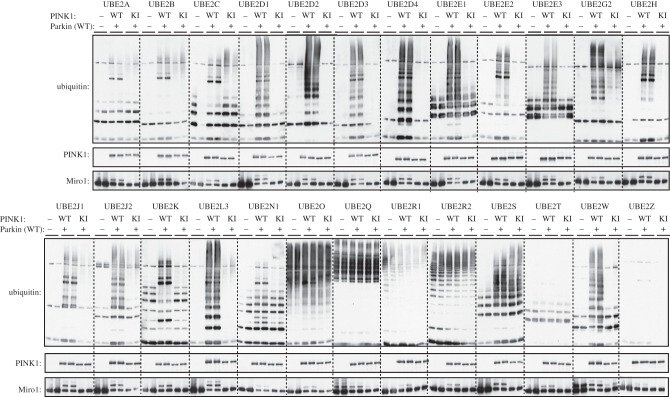
Parkin can interact with multiple different E2 conjugating enzymes to catalyse Miro1 ubiquitylation with or without free ubiquitin chain formation. An E2 scan of 25 different E2 conjugating enzymes was undertaken. Two micrograms of wild-type Parkin was incubated with 1 μg of wild-type (WT) or kinase-inactive (KI) (D359A) MBP-TcPINK in a kinase reaction for 60 min as described in §3. Activated Parkin was then added into pre-assembled ubiquitylation reactions containing 1 μg of the E2 conjugating enzyme as indicated. Reactions were terminated after 60 min by addition of SDS–PAGE loading buffer and resolved by SDS–PAGE. Miro1, ubiquitin and PINK1 were detected by immunoblotting using anti-SUMO, anti-FLAG and anti-MBP antibodies, respectively.

The identity of the physiological E2 that interacts with Parkin remains unknown. Previous studies have suggested that E2s play a critical role in controlling activity and specificity of RING E3 ligases, whereas the substrate specificity of HECT E3 ligases is conferred mainly via the E3–substrate interaction [46,47]. Parkin has previously been reported to partner with several ubiquitin-conjugating E2 enzymes, including UbcH7 (UBE2L3) [5,12], UbcH8 (UBE2L6) [48], UBC6 (UBE2J1) [49], UBC7 (UBE2G1, UBE2G2) [49] and Ubc13/Uev1a heterodimer (UBE2N/UBE2V1) [50]. Given that Parkin possesses both RING and HECT-like properties, it is not obvious how the nature of the ubiquitin conjugates would be influenced by the E2. We therefore decided to investigate how a panel of 25 E2 ligases impacted on the ability of Parkin to ubiquitylate Miro1 and induce formation of free polyubiquitin chains. This revealed that 23 of the 25 enzymes tested catalysed Miro1 ubiquitylation in a Parkin Ser^65^ phosphorylation-dependent manner (figure 4). Interestingly, these could be divided into two differential groups: one group of E2s catalysed robust free ubiquitin chain formation in addition to Miro1 ubiquitylation (UBE2D1, UBE2D2, UBE2D3, UBE2D4, UBE2E1, UBE2E3, UBE2J2, UBE2L3, UBE2N1 (weakly)), whereas the other group of E2s preferentially catalysed Miro1 ubiquitylation but no significant free ubiquitin chain formation (UBE2A, UBE2B, UBE2C, UBE2E2, UBE2G2, UBE2H, UBE2J1, UBE2K, UBE2O (weakly), UBE2R1, UBE2R2, UBE2S, UBE2T, UBE2Z (weakly)). In addition, two E2s catalysed Miro1 ubiquitylation in a Parkin-independent manner (UBE2Q and UBE2W).

### 4.5. Parkin disease-associated mutants exhibit differential effects on Parkin-mediated ubiquitylation

We next investigated the effect of Parkin disease-associated point mutations in the Miro1 substrate-based assay of E3 ligase activity. While the impact of mutations has been reported in previous studies, the majority of these have measured Parkin auto-ubiquitylation activity using either N-terminal-tagged versions of Parkin or N-terminally truncated forms of Parkin that do not contain the Ubl domain [10,11,28,29,51–54]. Consequently, the debate regarding the activity changes in disease mutants remains active with conflicting results being reported, e.g. Shimura *et al*. [51,52] have reported Arg42Pro (R42P) mutant to be ligase dead, whereas others have reported no effect on activity [10,11]. The same is true for Lys161Asn (K161N) mutation, which has been reported to be ligase inactivating [29,53] or having no effect on ligase activity [10,11,28].

**Figure 5.**
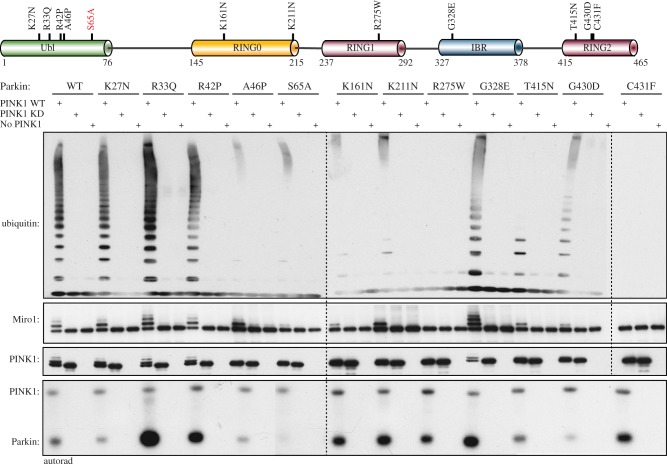
Heterogeneity of the effects displayed by Parkinson's disease-associated point mutations. (upper panel) A schematic of Parkin domain architecture showing the location of disease-associated Parkin mutants. (lower panel) Parkin mutants exhibit diverse effects on E3 ligase activity. Assays using wild-type (WT) and kinase-inactive (KI) PINK1 (D359A) in combination with WT and indicated mutants of Parkin and the substrate Miro1 were undertaken as described in §3. A kinase reaction including 0.1 mM [γ-^32^P] ATP (approx. 500 cpm pmol^−1^) was carried out in parallel for 60 min to confirm the phosphorylation as described in methods. Reactions were terminated after 60 min by addition of SDS loading buffer and resolved by SDS–PAGE. Miro1, Ubiquitin, Parkin and PINK1 were detected using anti-SUMO, anti-FLAG, anti-Parkin and anti-MBP antibodies, respectively. Representative of three independent experiments.

We therefore expressed full-length untagged Parkin encoding disease-associated point mutations spanning each domain of Parkin, namely: Lys27Asn (K27N), Arg33Gln (R33Q), R42P, Ala46Pro (A46P) (Ubl domain); K161N, Lys211Asn (K211N) (RING0 domain); Arg275Trp (R275W) (RING1 domain); Gly328Glu (G328E) (IBR domain); Thr415Asn (T415N); Gly430Asp (G430D); and Cys431Phe (C431F) (RING2 domain) (figure 5). We next assayed all of these mutants in parallel with wild-type and S65A Parkin to determine whether they exhibited differential ability to ubiquitylate Miro1 and/or form free ubiquitin chains after activation by PINK1 phosphorylation (figure 5). Diverse effects were observed, and the mutations could be classified into the following groups. Two mutants, C431F that disrupts the catalytic cysteine, and the RING1 mutant R275W, abolished Parkin activity against both Miro1 ubiquitylation and free ubiquitin chain formation (figure 5). The G430D mutation that lies adjacent to the catalytic cysteine caused a marked reduction in both free ubiquitin chain formation and Miro1 ubiquitylation as did the RING0 mutant K161N and the RING2 mutant T415N (figure 5). One group comprising the Ubl mutant A46P and the RING0 mutant K211N abolished free ubiquitin chain formation, but Miro1 ubiquitylation remained relatively intact although both mutants appeared to promote Miro1 monoubiquitylation rather than multi-monoubiquitylation (figure 5). Furthermore, two mutants exhibited differential increase in Parkin E3 ligase activity: both the Ubl mutant R33Q and the IBR mutant G328E led to increased Miro1 ubiquitylation, but only R33Q mutation also led to increased free chain formation. This effect might be explained by observation that the phosphorylation of R33Q and G328E mutants was significantly higher than that of wild-type Parkin (figure 5). Finally, one group comprising Ubl domain mutants K27N and R42P had no effect on Parkin-mediated ubiquitylation. No mutants led to a decrease/abolition of Miro1 ubiquitylation while maintaining the ability for free ubiquitin chain formation.

We have previously observed that following activation of Parkin by phosphorylating Ser^65^, MBP-PINK1 undergoes ubiquitylation that can be observed in a bandshift detectable by anti-ubiquitin immunoblotting and that is absent when Parkin is incubated in the presence of kinase-inactive TcPINK1 or using the S65A Parkin [15]. We have mapped the site of ubiquitylation to a lysine residue on MBP (Lys306 lying in the SYEEELVKDPR sequence motif; data not shown). We observed that MBP-PINK1 ubiquitylation was lost in mutants that led to decrease in Miro1 ubiquitylation or free chain formation (A46P, K211N) or both (S65A, R275W, C431F, G430D, K161N, T415N; figure 5) and was unaltered in mutants that had no effect or increased Parkin activity (WT, K27N, R33Q, R42P, G328E; figure 5).

### 4.6. Phosphorylation of Ser^65^ promotes discharge of ubiquitin from UbcH7-loaded E2 ligase: impact of disease-associated point mutations

**Figure 6.**
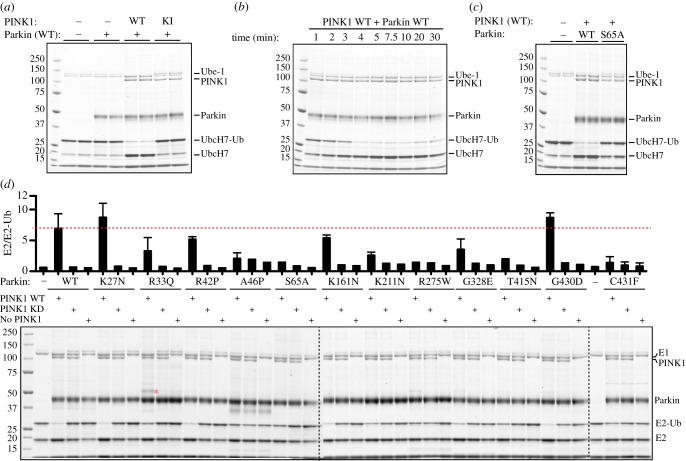
PINK1-dependent phosphorylation of Parkin Ser^65^ is required for discharge of ubiquitin from E2. Parkin was phosphorylated using wild-type (WT) or kinase-inactive (KI) MBP-TcPINK1. An E2 discharge assay was established by incubation of this mixture with 2 μg of UbcH7 that had been pre-incubated with 0.5 µg of E1 and FLAG-ubiquitin in the presence of ATP for 60 min. Reactions were allowed to continue for 15 min (*a,c,d*) or as indicated (*b*) and stopped using SDS–PAGE loading buffer in absence of reducing agent. Samples were resolved by SDS–PAGE and proteins detected by Colloidal Coomassie staining. (*a*) Ubiquitin-loaded UbcH7 (UbcH7-Ub) was observed in the absence of Parkin (lanes 1,2). WT Parkin only in the presence of WT MBP-TcPINK1 was able to efficiently discharge UbcH7-Ub (lanes 5,6). No discharge was observed with WT Parkin alone (lanes 3,4) or WT Parkin in the presence of KI MBP-TcPINK1 (lanes 7,8). (*b*) Time course of E2 discharge after addition of activated WT Parkin in the presence of WT MBP-TcPINK1 demonstrated rapid and maximal discharge of UbcH7-Ub at 4 min. (*c*) Abrogation of UbcH7-Ub discharge by Parkin Ser65Ala (S65A; lanes 5,6) in contrast to WT Parkin in the presence of WT PINK1 (lanes 3,4). (*d*) Comparison of the effects Parkin disease mutations on ubiquitin discharge from UbcH7. Red dotted line indicates the WT activity. K27N, R33Q, R42P, K161N, G430D and G328E mutants showed no significant changes in activity. A46P, S65A, K211N, R275W, T415N and C431F displayed markedly decreased E2-ubiquitin discharge ability. Asterisk indicates the R33Q Parkin–ubiquitin thioester. Representative of three independent experiments.

We next investigated the mechanism of Parkin activation upon PINK1-dependent phosphorylation of Parkin at Ser^65^. Given the direct interaction of the Ubl domain with the RING1 domain [29], we hypothesized that Ser^65^ phosphorylation might influence binding of ubiquitin-loaded E2 to the RING1 and/or E2-mediated ubiquitin transfer. We therefore investigated whether phosphorylation of Parkin Ser^65^ influences its ability to induce discharge of ubiquitin from a ubiquitin-loaded E2, UbcH7 (E2-Ub). To load UbcH7 with ubiquitin, we incubated E1 (UBE1), UbcH7, and ubiquitin in the presence of Mg-ATP for 60 min at 30°C. Non-phosphorylated or TcPINK1-phosphorylated Parkin was then added to the reaction mixture for 15 min. Reactions were terminated using LDS loading dye and immediately analysed by electrophoresis on a polyacrylamide gel that was subsequently stained with Coomassie. This analysis enabled the facile discrimination of ubiquitin-conjugated UbcH7 from non-conjugated UbcH7. Wild-type non-phosphorylated Parkin (in the absence of PINK1 and in the presence of kinase-inactive TcPINK1) failed to mediate significant discharge of ubiquitin from UbcH7; however, Parkin that was phosphorylated by wild-type TcPINK1 induced a robust ubiquitin discharge illustrated by reduction of UbcH7–Ub thioester band (figure 6*a*). We did not observe any Parkin–ubiquitin thioester, which is consistent with previous analysis of full-length Parkin [4,5,32]. A time-course analysis revealed that under the conditions used maximal ubiquitin discharge induced by PINK1-phosphorylated Parkin occurred within 4–5 min (figure 6*b*). Consistent with the requirement of phosphorylation by PINK1, the Parkin Ser65Ala mutation prevented ubiquitin discharge from UbcH7 (figure 6*c*).

We next investigated the effects of disease-associated mutations on the ubiquitin discharge from UbcH7 after activation by TcPINK1. Parkin mutants that exhibited normal or increased ubiquitylation of Miro1, namely K27N, R33Q, R42P and G328E, showed no significant changes in the ubiquitin discharge ability (figure 6*d*). Strikingly, we observed a Parkin–ubiquitin thioester for the R33Q mutant, suggesting that this mutation may lead to conformational changes that render the complex more stable when compared with wild-type Parkin (figure 6*d*).

Parkin mutants A46P, R275W and T415N were similar to the S65A mutant and the catalytic active site disease mutant C431F and showed significantly reduced E2-ubiquitin discharge ability, suggesting that these residues are required for efficient ubiquitin discharge upon Parkin Ser^65^ phosphorylation and E2 binding to Parkin. The remaining mutants comprising RING0 mutants K161N, K211N and RING2 mutant G430D exhibited intact or modestly reduced (K211N) Parkin phosphorylation-dependent E2 discharge.

## 5. Discussion

This study provides fundamental evidence that PINK1 phosphorylation at Ser^65^ activates Parkin E3 ligase. Most importantly, by elaborating novel *in vitro* assays to assess Parkin activity, we demonstrate that phosphorylation of Ser^65^ by PINK1 is critical to enable Parkin to ubiquitylate its substrate Miro1 and induce formation of free ubiquitin chains (figure 1). Importantly, this is dependent on full-length Parkin, because ΔUbl-Parkin failed to ubiquitylate Miro1 (figure 2). This suggests that phosphorylation at Ser65 may not act exclusively in relieving autoinhibition but may also have an additional role in Parkin activation. The importance of the Ubl domain in Parkin activation is also underscored by a recent study in which it was observed that ΔUbl-Parkin prevented formation of a Parkin C431S oxyester in cells in response to mitochondrial depolarization [55]. Using an E2-ubiquitin discharge assay, we demonstrated that Ser^65^ phosphorylation of Parkin is critical for efficient discharge of ubiquitin from the UbcH7 E2 ligase (figure 6*a–d*). Furthermore, we provide new mechanistic insights into the pathogenicity of human disease-associated mutations of Parkin (summarized in the electronic supplementary material, table S1).

### 5.1. Miro1 is a direct Parkin substrate

Our study is the first to show that Miro1 is a direct substrate of Parkin *in vitro*. Two previous studies suggested that the levels of Miro1 may be regulated by PINK1 and Parkin, but results were conflicting. Wang *et al*. [44] reported that overexpression of PINK1 and/or Parkin led to decreased Miro1 levels in HEK 293T cells, and the authors reported that this was mediated by PINK1-dependent phosphorylation of Miro1 at Ser^156^. On the other hand, Liu *et al.* [43] found no evidence for phosphorylation at Miro1 Ser^156^ and found Miro1 levels were lower in PINK1 siRNA-targeted HeLa cells as well as in PINK1 KO MEF cells compared with wild-type MEF cells. We have not been able to phosphorylate the Miro1 (1–592) fragment that lacks the transmembrane domain with TcPINK1 (data not shown).

A recent global ubiquitylation analysis of Parkin-regulated proteins reported Miro1 Lys^572^, Lys^153^, Lys^194^ and Lys^235^ ubiquitylation in cells overexpressing tagged Parkin stimulated with CCCP; however, it did not address whether Parkin catalysed the ubiquitylation of these sites directly [32]. In our assay, the Lys572Arg mutant drastically reduced ubiquitylation as judged by Coomassie staining analysis, suggesting that Lys^572^ is a major site targeted by Parkin (the electronic supplementary material, figure S3). While we also identified Lys^153^, Lys^230^, Lys^235^ and Lys^330^ as direct sites of Parkin ubiquitylation, we cannot rule out additional sites such as Lys^194^, which may be of lower stoichiometry (figure 3). Miro1 plays a crucial role in mitochondrial trafficking by tethering mitochondria to KIF5 motor proteins, enabling mitochondria to be transported along microtubules [56]. Several of the sites we have identified lie within or near functional domains of Miro1, including Lys^153^ that is located within the N-terminal GTPase domain of Miro1, Lys^330^ that lies within the second EF hand domain and Lys^572^ that lies in a C-terminal linker region near its transmembrane domain that localizes Miro1 to the outer mitochondrial membrane. It would be exciting to test whether Parkin-mediated ubiquitylation of Miro1 leads to alteration of its GTPase activity, localization, calcium binding or role in mitochondrial transport.

The finding that PINK1-activated Parkin induces multi-monoubiquitylation of Miro1 rather than attachment of a polyubiquitin chain highlights the potential diversity of Parkin's catalytic activity. Previous studies that have used Parkin with activating N-terminal tags have also observed monoubiquitylation activity. For example, Tanaka's laboratory first reported that Parkin could catalyse monoubiquitylation *in vitro* using a pseudo-substrate assay in which MBP-fused Parkin targeted residues within MBP in *cis* [11]. Multi-monoubiquitylation activity has also been reported in an auto-ubiquitylation assay using GST-Parkin [10]. Future work expanding our initial analysis to test a variety of reported substrates of Parkin, including mitochondrial proteins such as VDAC1 and Tom70 as well as non-mitochondrial proteins, e.g. CDCrel1, Pael-R and PARIS [33], will be crucial in determining the mechanistic qualities and functional effects of ubiquitylation by Parkin. Monoubiquitylation of substrates has previously been shown to be important for histone regulation, DNA repair and viral budding, whereas multi-monoubiquitylation has been implicated in endocytosis [57,58]. The consequence of mono/multi-monoubiquitylation of outer mitochondrial membrane proteins such as Miro1 is unknown. It could be critical for intermolecular signalling at the mitochondria, because, in more well-studied systems such as endocytosis, many proteins, e.g. Eps15, contain ubiquitin interaction motifs that bind monoubiquitin [59]. Alternatively, it is possible that monoubiquitylation of Miro1 and other Parkin substrates targets these for polyubuitylation chain extension by other E3 ligases. This has been demonstrated for the ubiquitylation of proliferating cell nuclear antigen which binds DNA during DNA replication [60]. However, the possibility that Parkin itself can catalyse the formation of polyubiquitylated Miro1 under specific cellular conditions or in the presence of a regulatory protein missing from our assay cannot be excluded, e.g. the E4 CHIP has previously been shown to enhance Parkin-mediated polyubiquitylation of the substrate Pael-R [61]. In future, it would be critical to establish whether Miro1 is multi-monoubiquitylated or polyubiquitylated *in vivo* and in the latter case to investigate whether other E3 ligases are required to achieve this.

### 5.2. Analysis of Parkin disease mutants

Biochemical and structural studies of the RBR E3 ligases HOIP and HHARI have provided strong evidence that RBR ligases can undergo an intermediate ubiquitin–thioester state between a highly conserved RING2 domain cysteine and ubiquitin [5,6,62,63]. The recent structures of Parkin strongly predict that Cys^431^ within the RING2 domain is likely to be the active site cysteine that would form a thioester [27–29]. An intermediate oxyester of Parkin has been demonstrated in mammalian cells, with two groups having successfully trapped ubiquitin using a C431S mutant Parkin after stimulation of cells with the mitochondrial uncoupler CCCP [55,64]. By contrast, direct experimental observation of a thioester-intermediate state for full-length human Parkin has been elusive [4,5], with the strongest evidence to date obtained by analysis of the isolated *Drosophila* Parkin IBR-RING2 domain in which it was demonstrated that the homologous residue Cys^449^ formed a thioester that was abolished by the Cys449Ala mutant [4]. Our finding that the C431F disease mutant abolishes Parkin E3 ligase activity in a substrate assay (figure 5) lends further support to the essential catalytic role of cysteine Cys^431^ and is consistent with previous analysis of the C431F mutant in auto-ubiquitylation assays that also found no E3 ligase activity [11,53,54]. Furthermore, we found that the C431F mutant significantly abrogated ubiquitin discharge from the E2-ligase UbcH7 (figure 6*d*), and this is in agreement with other RBR family enzymes wherein mutation of the catalytic cysteine to alanine also prevented E2-ubiquitin discharge such as the Cys357Ala mutation in HHARI [6] and the Cys885Ala mutation in HOIP [62]. Under our assay conditions, we did not observe an intermediate ubiquitin–thioester state for activated full-length wild-type Parkin (figure 6*d*), similar to other groups who have studied full-length Parkin [4,5]. However, we unexpectedly were able to trap a ubiquitin–thioester by the Parkin R33Q mutant in our assay (figure 6*d*), which represents the first experimental demonstration of the existence of a thioester-intermediate state for recombinant full-length Parkin (figure 6*d*).

Importantly, our analysis is the first to reveal that the R275W mutant leads to complete abolition of Parkin E3 ligase activity (figure 5). This mutant has been the subject of intense investigation, because it was identified as a compound heterozygote mutation in a family with evidence of Lewy body pathology at post-mortem [65]. While Arg^275^ lies within the RING1 helical domain core, the nature of its pathogenicity was unknown, because all previous studies of the R275W mutant had found no effect on E3 ligase activity [10,11,54]. Furthermore, we observed that the R275W mutant significantly reduced ubiquitin discharge from the E2 (figure 5*d*). Given that R275W is in the RING1 domain, this might suggest that it disrupts E2 binding to the RING1 domain or transfer of ubiquitin from the loaded E2 onto the ubiquitin acceptor Cys^431^ on the RING2 domain, or equally both these steps.

Lysine161 (Lys^161^) lies within the RING0 domain, and structural analysis suggests that it forms a salt bridge with the RING2 domain as well as a putative phosphopeptide binding pocket that may be important for Parkin activation [28]. Consistent with this hypothesis, we have found that the K161N mutant leads to a significant decrease in the E3 ligase activity of Parkin towards both Miro1 ubiquitylation and free chain formation (figure 5). The effect of this mutant on E3 ligase activity has been widely debated, because most studies using auto-ubiquitylation assays have found no effect on Parkin E3 ligase activity [10,11,28,66] whereas a few studies found that K161N mutants exhibited lower activity [29,53]. We found that this mutant did not affect E2-ubiquitin discharge, suggesting an alternative mechanism for disruption of ubiquitylation. It will be exciting to test whether Lys^161^ indeed forms a phosphopeptide binding pocket and whether this binds a Ser^65^-phosphorylated peptide leading to activation.

An unexpected discovery from our mutation analysis was the differential effect of the K211N and A46P mutants on Parkin E3 ligase activity with abolition of free chain ubiquitylation but with relative preservation of the Miro1 substrate ubiquitylation (figure 5). Such disease-associated mutants or artificial mutants have not been reported before for either Parkin or other members of the RBR E3 ligase family of enzymes. Lysine211 (Lys^211^) lies within the RING0 domain and has also been suggested to form a putative phosphopeptide binding pocket [28], whereas Ala46 (Ala^46^) lies within the Ubl domain. Previously, the K211N mutant has been reported to have no effect on E3 ligase activity using auto-ubiquitylation assays [10,11], and the A46P mutant was reported to be hyperactive in an auto-ubiquitylation assay [12]. Interestingly, both the A46P and K211N mutants led to a significant decrease in E2-ubiquitin discharge (figure 6*d*) suggesting that E2 binding and ubiquitin discharge may be essential for the formation of the free ubiquitin chains, but are dispensable for the catalytic activity of Parkin directed towards Miro1 ubiquitylation at least for these mutant forms of Parkin.

Two mutants, R33Q and G328E, exhibited evidence of increased Parkin E3 ligase activity upon phosphorylation by PINK1. Previously, the Ubl domain mutants, including R33Q, K27N and R42P, have been found to be constitutively hyperactive in auto-ubiquitylation assays [12]. Similarly, we observed low basal auto-ubiquitylation activity for R33Q compared with none for wild-type Parkin in the absence of PINK1 (data not shown). Furthermore, upon phosphorylation by PINK1, the R33Q mutant displayed increased activity towards Miro1 ubiquitylation and polyubiquitin chain formation (figure 5). This was associated with striking increase in phosphorylation of R33Q compared with wild-type Parkin (figure 5). The arginine33 (Arg^33^) residue is located within the α1 helix which forms extensive interactions with a beta-sheet comprising strands 2, 1 and 5 (from N- to C-terminus) and contributes to the integrity of the Ubl domain. Mutation of R33 to glutamine would disrupt a stabilizing hydrogen bond between R33 and the adjacent residue, Q34. Molecular dynamics simulations of the R33Q mutation in murine Ubl domain [67] predict that loss of this hydrogen bond would lead to decreased stability of the α1 helix, and as a result increased structural fluctuations in the β 2, 1, 5-sheet. Serine 65 is located at the N-terminus of β5 and in all structures of the Ubl domain is partially buried; it is plausible that fluctuations in this strand induced by the R33Q mutation may lead to greater surface exposure of Ser^65^, increased accessibility and phosphorylation by PINK1 and a subsequent increase in E3 ligase activity (the electronic supplementary material, figure S4). However, enhanced phosphorylation of the R33Q mutant was not associated with an increase in UbcH7 discharge of ubiquitin (figure 6*d*) suggesting an alternative mechanism for increased activity.

Previous reports on the effect of the G328E mutant on E3 ligase activity have been more controversial, with several groups reporting no change in E3 ligase activity [10,11,54] whereas one report suggested a decreased activity in auto-ubiquitylation assays [29]. The G328E mutant stimulated Miro1 ubiquitylation without any effect on the formation of free ubiquitin chains. Similar to R33Q, this was not associated with any significant change in the E2 discharge of ubiquitin (figure 6*d*). One explanation for the lack of effect of G328E on free chain formation is that the Gly^328^ residue that is located in the RING1 : IBR interface may directly interact with the substrate Miro1 and the G328E mutant may stabilize this interaction leading to enhanced Miro1 ubiquitylation. In future studies, it would be important to determine whether the G328E mutant enzyme has a higher affinity for its substrate.

### 5.3. Analysis of Parkin–E2 interactions

An important question remaining in the field is establishing the identity of the physiological E2(s) that Parkin interacts with to catalyse ubiquitylation of its substrates. Extensive analyses of RING E3 ligases indicate that the diversity and specificity of ubiquitin conjugates is significantly influenced by partnering E2s. In contrast, for HECT E3 ligases the pattern of ubiquitin conjugates appears to be largely independent of the identity of the E2 and conferred principally by the E3–substrate interaction. Given that Parkin possesses both RING- and HECT-like properties, it is not obvious how the nature of the ubiquitin conjugates would be influenced by the E2 [46,68]. We investigated how a panel of E2 ligases impacted the ability of Parkin to monoubiquitylate Miro1. Previous studies identified UbcH7 (UBE2L3), UbcH8 (UBE2L6), UBC6 (UBE2J1), UBC7 (UBE2G1, UBE2G2) and Ubc13/Uev1a heterodimer (UBE2N/UBE2V1) [33,64,69,70] as partnering E2s for tagged Parkin. Our assay enabled us to determine the specificity of E2 conjugating enzymes that control ubiquitylation of Miro1 by Parkin in the presence or absence of Ser^65^ phosphorylation. It also permitted us to investigate whether E2s played any role in determining the pattern of ubiquitin conjugates that are formed by Parkin in our assay. We tested 25 different E2s and strikingly observed that the ubiquitylation of the substrate Miro1, by Parkin, was not influenced by the vast majority of the E2s tested. This observation suggests that Parkin exhibits mainly HECT-like properties in which the interaction between Parkin and Miro1 is the critical feature governing the ubiquitylation of Miro1. We nevertheless did observe a differential effect of E2s in their ability to enable Parkin to catalyse the formation of free ubiquitin chains, e.g. UBE2L3 enabled both Miro1 ubiquitylation and free chain formation, whereas UBE2H only enabled Parkin to mediate Miro1 ubiquitylation. This does suggest that the formation of free chains may be critically dependent on the E2–E3 interaction. The molecular mechanisms underpinning E2-mediated ubiquitin chain formation remain poorly understood except for a few examples. In yeast, the anaphase promoting complex/cyclosome (APC) has been shown *in vitro* to promote multi-monoubiquitylation of cyclin B in the presence of Ubc4, whereas Ubc1 enables it to catalyse polyubiquitin chain formation [71]. Moreover, APC exploits the differential selectivity of both E2s to target substrates for polyubiquitylation *in vivo* [71]. It would be interesting to study a RING2 HECT-defective mutant and repeat the E2 screen to identify any E2s in which the ability to generate ubiquitin free chains was dependent on the E2–RING1 interaction alone. It will also be important to determine the universality of our findings and test the E2 specificity for other Parkin substrates we identify as being regulated by Ser^65^ phosphorylation. Our data suggest that *in vitro* analysis is unlikely to be helpful in pinpointing physiological E2 ligases that act with Parkin. To address this question, in future, *in vivo* approaches such as genetic screens could be used.

## 6. Summary

Overall, we have elaborated for the first time in the field a substrate-based assay of untagged full-length Parkin. This has revealed the critical importance for Ser^65^ phosphorylation by PINK1 for Parkin activation and provided further evidence that Miro1 is a bona fide substrate of Parkin. Our studies reveal a critical requirement for the Ubl domain in substrate ubiquitylation and also demonstrate that Ser^65^ phosphorylation by PINK1 stimulates ubiquitin discharge from the E2 ligase UbcH7 that provides one explanation of how phosphorylation might activate Parkin E3 ligase activity.

The assay and reagents we have developed will be extremely valuable in addressing critical questions of Parkin biology. As proof of concept, we have deployed these to understand how Parkin disease mutations impact on catalytic activity, and this has revealed new fundamental knowledge on the mechanism of pathogenicity of these mutations. Recently, novel regulatory modifications have been reported for Parkin E3 ligase activity, including c-Abl-induced tyrosine phosphorylation [72] and cysteine sulfhydration [73]; it will be interesting to test the influence of these modifications on Parkin activity in our assay. Lastly, there is increasing interest to explore the therapeutic potential of developing small molecule activators of Parkin E3 ligase that could have the potential to treat PD. The assay we have developed to measure ubiquitylation of Miro1 or discharge of ubiquitin-loaded UbcH7 could serve as a basis for setting up a screen to identify compounds that activate Parkin.

## Supplementary Material

Supplementary Figures
