# Supplementary material for: Phosphorylation of Parkin at Serine65 is essential for activation: elaboration of a Miro1 substrate-based assay of Parkin E3 ligase activity
Source: Open Biol. 2014 Mar 19;4(3):130213. doi: 10.1098/rsob.130213 (PMC3971407; doi:10.1098/rsob.130213)
Supplement: Supplementary Figures [file rsob130213supp1.pdf]

Supplemetary Figure 1

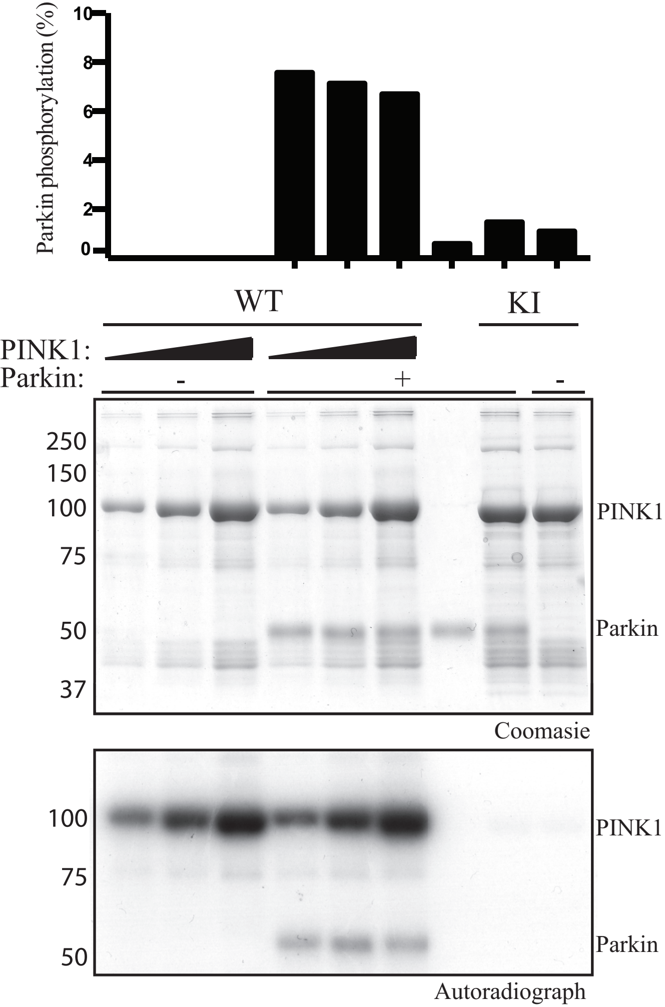

Supplemetary Figure 2

Site	Peptide sequence	Theoretical m/z	Observed m/z	Ppm error	Charge state	Mascot score
K6	GGMQIFV**K**TLTGK	498.6097	498.6129	6.46	3	24
K11	TLTG**K**TITLEVEPSDTIENVK	801.4269	801.4301	4.05	3	42
K27	TITLEVEPSDTIENV**K**AK	701.039	701.0391	0.26	3	25
K33	IQD**K**EGIPPDQQR	819.4157	819.4151	-0.75	2	18
K48	LIFAG**K**QLEDGR	730.8964	730.8994	4.01	2	63
K63	TLSDYNIQ**K**ESTLHLVLR	748.7376	748.7445	9.24	3	32

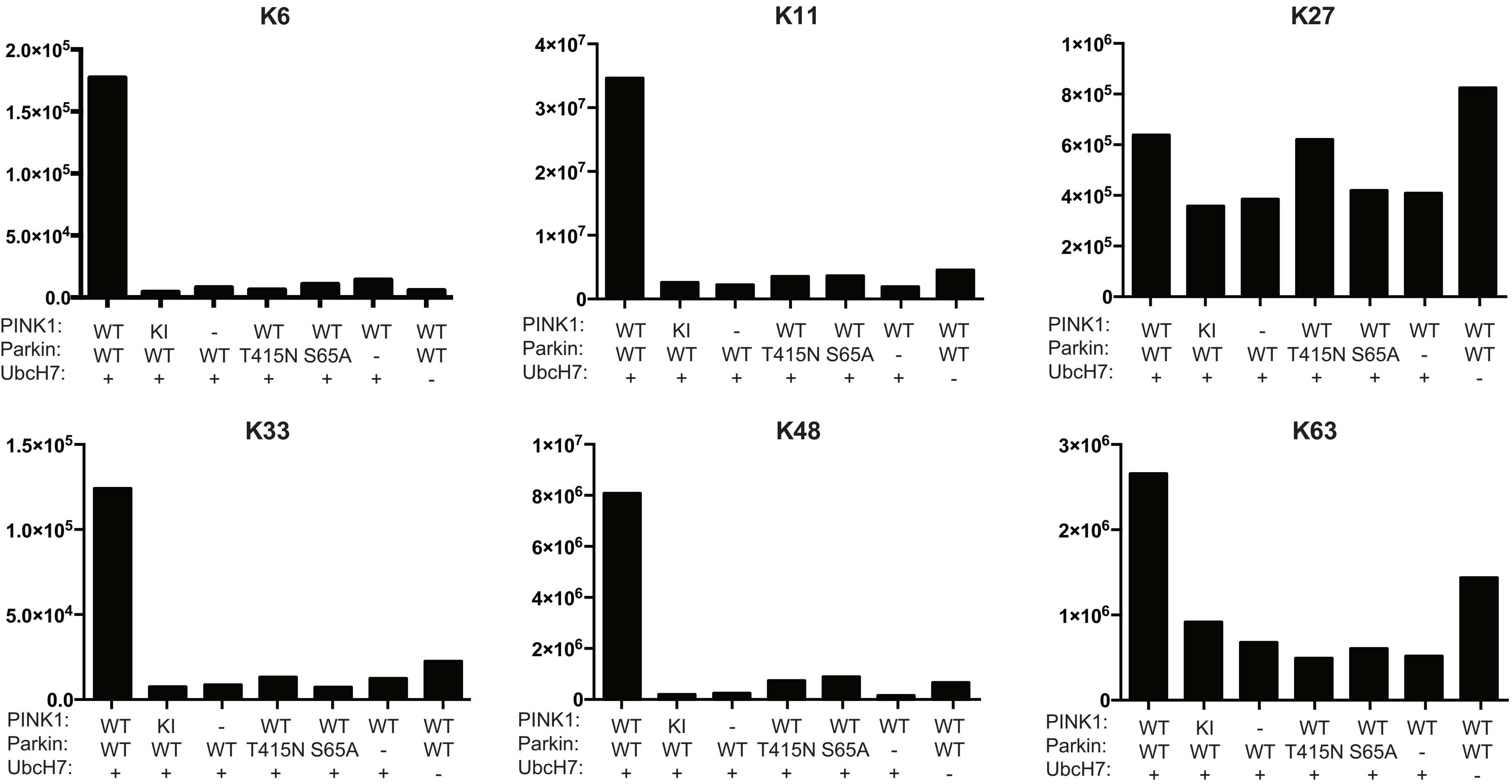

Supplemetary Figure 3

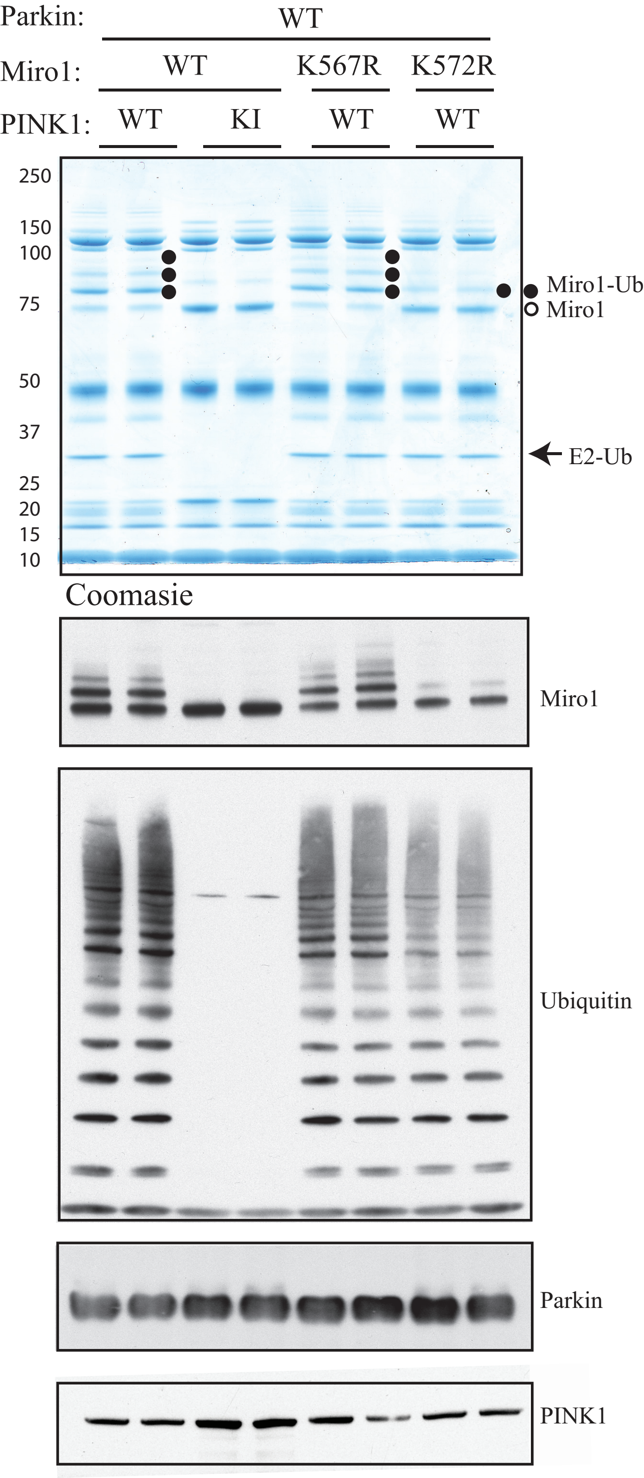

Supplemetary Figure 4

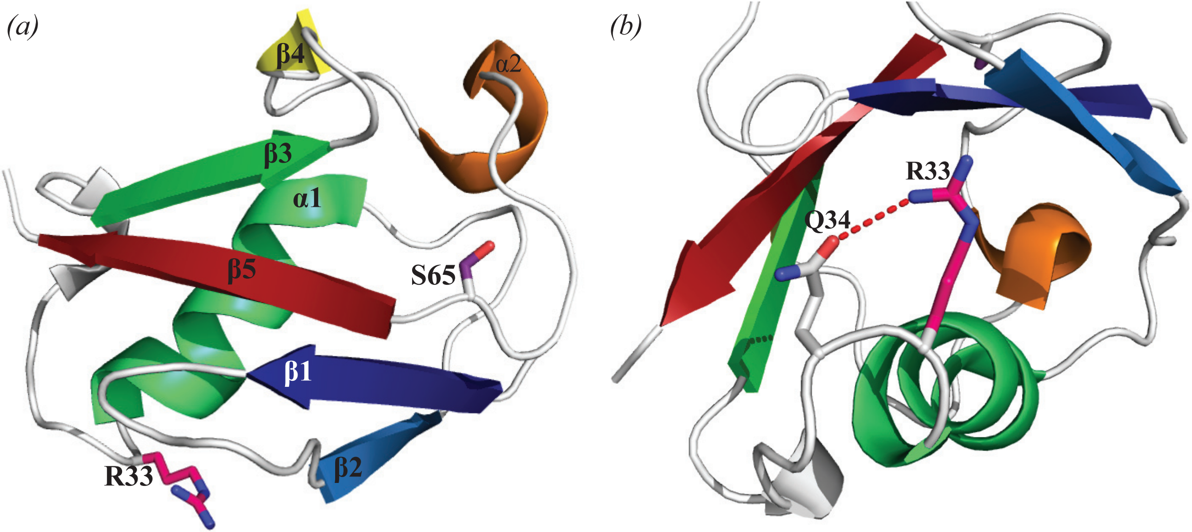

Supplementary Table 1

Parkin mutation	Location	E3 activity		E2 discharge
		Miro1-Ub	Free-chains	
WT	N/A	↔	↔	↔
K27N	Ubl	↔	↔	↔
R33Q	Ubl	↑	↑	↔
R42P	Ubl	↔	↔	↔
A46P	Ubl	↔	No	↓
S65A	Ubl	No	No	↓
K161N	RING0	↓	↓	↔
K211N	RING0	↔	No	↔
R275W	RING1	No	No	↓
G328E	IBR	↑	↔	↔
T415N	RING2	↓	↓	↓
G430D	RING2	↓	↓	↔
C431F	RING2	No	No	↓
